# Prevalence of *Blastocystis* sp. infection in several hosts in Brazil: a systematic review and meta-analysis

**DOI:** 10.1186/s13071-020-3900-2

**Published:** 2020-01-14

**Authors:** Andernice dos Santos Zanetti, Antonio Francisco Malheiros, Tatiane Amorim de Matos, Fabiana Gulin Longhi, Luciana Melhorança Moreira, Samuel Laudelino Silva, Solange Kimie Ikeda Castrillon, Silvana Margarida Benevides Ferreira, Eliane Ignotti, Omar Ariel Espinosa

**Affiliations:** 1 Post-Graduation Program in Environmental Science, Faculty of Agricultural and Biological Sciences, State University of Mato Grosso (UNEMAT), Cáceres, Mato Grosso Brazil; 2 The Brazilian Centre for Evidence-based Healthcare: A Joanna Briggs Institute Centre of Excellence, São Paulo, São Paulo Brazil; 3 Faculty of Agricultural and Biological Sciences, State University of Mato Grosso (UNEMAT), Cáceres, Mato Grosso Brazil; 4 Post-Graduation Program in Nursing, Federal University of Mato Grosso (UFMT), Cuiabá, Mato Grosso Brazil; 5 Department of Nursing, Faculty of Health Sciences, State University of Mato Grosso (UNEMAT), Cáceres, Mato Grosso Brazil; 6 Department of Medicine, Faculty of Health Sciences, State University of Mato Grosso (UNEMAT), Cáceres, Mato Grosso Brazil

**Keywords:** *Blastocystis*, *Blastocystis* infection, Meta-analysis, Brazil, Systematic review

## Abstract

**Background:**

*Blastocystis* sp. affects a wide variety of animals and is the most common protozoan in human fecal samples with potential pandemic distribution. In the present study, a systematic review and meta-analysis were conducted to determine the prevalence and distribution of *Blastocystis* sp. in different classes of hosts in Brazil.

**Methods:**

Studies that analyzed hosts of various classes, including humans, domestic animals, wild animals or captive animals, were considered. The pooled prevalence of *Blastocystis* sp. infection was estimated by random effects models.

**Results:**

For humans, similar prevalence rates were found for males (31.0%, 95% CI: 17.0–45.0%; weight 10%) and females (28.0%, 95% CI: 16.0–41.0%; weight 10%); the state of Mato Grosso do Sul showed the highest prevalence, with 41.0% positivity (95% CI: 36.0–46.0%; weight 2.9%). The prevalence among immunocompromised patients was 5.0% (95% CI: 3.0–7.0%; weight 10%), and the most common cause of immunosuppression was hemodialysis, with 23.0% (95% CI: 17.0–29.0%; weight 12.4%). Among classifications according to interaction with humans, wild and domestic animals presented values of 19.0% (95% CI: 7.0–31.0%; weight 42.6%) and 17.0% (95% CI: 13.0–21.0%; weight 29.6%), respectively. Among these animals, mammals (39.0%, 95% CI: 21.0–56.0%; weight 47.3%) and birds (18.0%, 95% CI: 10.0–27.0%; weight 39.3%) exhibited the highest prevalence. Phylogenetic analysis of *Blastocystis* sp. revealed greater genetic diversity for clades of subtypes (STs) ST1, ST2 and ST3.

**Conclusions:**

The overall prevalence of *Blastocystis* sp. in the Brazilian human population was 24%, which reflects the reality in the South, Southeast and Midwest regions, where prevalence rates of up to 40% were found. Among animals, mammals and birds exhibited the highest prevalence.
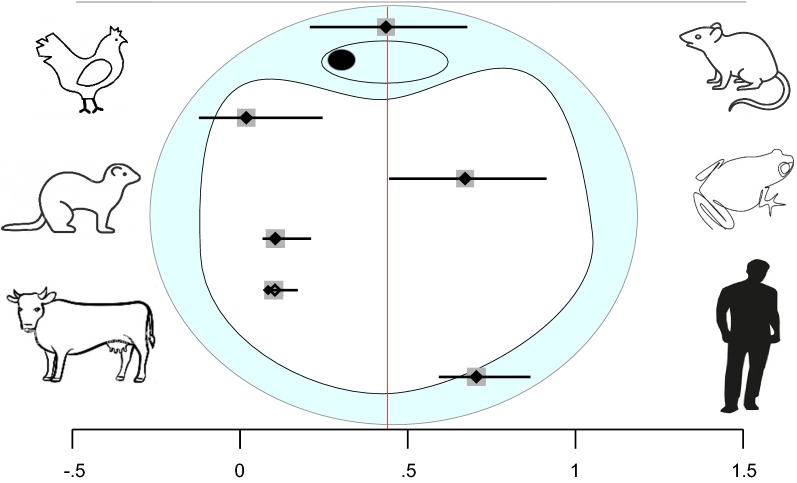

## Background

*Blastocystis* sp. is a protozoan parasite with no flagella belonging to class Blastocystea. This protozoan is commonly found in the gastrointestinal tracts of humans in addition to a wide variety of animals of various classes [1, 2].

Transmission of *Blastocystis* sp. occurs through the fecal-oral route [3]. Several studies suggest that contamination of water with fecal matter may be a source of infection [4–6]. For this reason, this parasite was included in water sanitation programmes and the Health Programme of the World Health Organization (WHO) [7].

*Blastocystis* sp. has been described as the most common eukaryotic organism in human fecal samples. It has a potential pandemic distribution, presenting prevalence rates that vary widely between countries and even between regions of the same country, reaching 30% in developed countries and up to 76% in developing countries [8–13].

There is an ongoing debate about the commensal or pathogenic nature of *Blastocystis* sp. In recent decades, several epidemiological studies have shown *in vitro* evidence in animal models and in humans strongly suggesting the pathogenic potential of this parasite [14–19]. Correlations between pathogenicity and subtypes (STs) of *Blastocystis* sp. have also been the subject of research, and results have indicated that not all strains of a given subtype are pathogenic. This fact suggests that subtype is not the only factor related to the pathogenicity of this parasite [15, 16]. Furthermore, not all humans are susceptible to infections caused by *Blastocystis* sp., which can be detected in asymptomatic hosts [1]. Regardless, there is a growing recognition of the pathogenicity of *Blastocystis* sp. in humans, even though its virulence mechanisms are not well described, because the symptoms of infection by this parasite are associated with non-specific gastrointestinal symptoms such as diarrhea, nausea, vomiting, abdominal pain and irritable bowel syndrome [20, 21].

*Blastocystis* sp. is commonly reported as a clinically relevant infection among immunosuppressed individuals that can result in severe diarrhea due to the progressive decline in defense mechanisms of these patients. Data on the prevalence of this pathogen indicate rates ranging from 15 to 25% in hemodialysis patients and from 20 to 35% in renal transplant patients [22–26].

Regarding *Blastocystis* sp. STs, analyses of the small subunit of the ribosomal RNA gene (*SSU* rDNA) have revealed genetic diversity represented by 17 genetically distinct strains (ST1-ST17) [27, 28]. To date, 10 subtypes have been found in humans (ST1-ST9 and ST12); however, 90–95% of human infections can be attributed to one of the ST1-ST4, with a predominance of ST3 [29–33]. All subtypes found in humans, except for ST9, have also been identified in animals, including non-human primates, mammals and birds [34, 35]. In Brazil, the subtypes found in animal hosts, including domestic, wild and captive, were ST1-ST5 and ST8 among mammals and birds [36, 37], indicating the potential for zoonotic transmission.

ST10 to ST17 have been found exclusively in animal hosts, non-human primates and other mammals [1, 27, 31, 38, 39]. Potential STs in non-mammal and non-avian hosts, so-called NMASTs (non-mammal and non-avian STs), have also been proposed for amphibians, reptiles and insects [39, 40].

Although there are data regarding the prevalence of *Blastocystis* sp. in some regions, no analysis of pooled prevalence and distribution according to STs by geographical area, type of host, sex and immunosuppression in Brazil has been published to date. This pioneering systematic review aimed to understand the prevalence and distribution of *Blastocystis* sp. in different classes of hosts in Brazil.

## Methods

The protocol for this systematic review was published in the International Prospective Register of Systematic Reviews (PROSPERO 2018: CRD42018116792) before its implementation, as described in Additional file 1: Text S1. The protocol and final report were developed based on the Cochrane Handbook for Systematic Reviews of Interventions [41].

### Review question

What is the prevalence, geographical distribution and phylogenetic relationships of *Blastocystis* sp. subtypes parasitizing different host species in Brazil?

### Inclusion criteria

This review considered studies conducted with several hosts of various classes, including humans and domestic, wild and captive animals, in Brazil to determine the prevalence and molecular identification of *Blastocystis* sp. subtypes through coprological analyses and molecular techniques, excluding those that did not report a positivity percentage.

### Search strategy

An initial search limited to MEDLINE was performed using MeSH index terms and related keywords. The search was followed by an analysis of the text of the title, abstract and index terms used to describe the article. A second search using all identified keywords and index terms was performed in all included databases. As a source of gray literature, a search was then performed in reference lists for dissertations that evaluated the prevalence of *Blastocystis* sp. Because this study focused on Brazil, the search was limited to articles published in English, Spanish and Portuguese. The search had no limits regarding the start date and was concluded in February 2019.

Studies were searched in the following databases: the Spanish Bibliographic Index of the Health Sciences (IBECS), the Latin American and Caribbean Health Sciences Literature (LILACS), the United States National Library of Medicine bibliographic database (Medline), the Elsevier database (EMBASE), the Cochrane Library, and National Institute for Health and Clinical Excellence (NICE). The MeSH index terms searched were *Blastocystis*, *Blastocystis* infections, Brazil, prevalence, and parasitology. The search terms are provided in Additional file 1: Text S1.

### Evaluation of methodological quality

The articles selected for data retrieval were analyzed by two independent reviewers to evaluate the methodological validity of each paper before being included in this review. We evaluated the quality of the included publications based on criteria from the Grading of Recommendations Assessment, Development and Evaluation (GRADE) method. The studies received one point if they did not have limitations in study design or execution (risk of bias), inconsistency of results, indirectness of evidence, imprecision and publication bias. A score of four to five points was considered high quality, three points was considered moderate quality, and two to zero points was considered low quality.

### Data extraction

The data were entered in Review Manager (RevMan 5.3) for analysis. A data extraction table was designed to evaluate the quality of the demographic data, study location, sample size, number of cases, number of positives and diagnostic test.

### Data synthesis

The meta-analysis random-effect model was applied to analyze the pooled prevalence, with a 95% confidence interval (CI), of *Blastocystis* sp. infection in both humans and animals. Heterogeneity among the studies was analyzed using the Higgins test (I^2^), which describes the percentage of total variation across studies that is due to heterogeneity rather than chance. Analyses were performed using Stata v. 13.1.

### Phylogenetic analysis

Partial sequences of the *SSU* rDNA gene from various *Blastocystis* sp. subtypes (ST1-ST5, ST8, ST11, ST12 and ST14) retrieved from the GenBank database were analyzed. Of the subtypes described in Brazil, only ST6 and ST7 were not included in the analysis because sequences of the *SSU* rDNA gene fragment were not available for these subtypes. The sequences were aligned in Clustal X software [42], with changes to the parameters related to the insertion of “Gaps” (insertion penalty = 1, extension penalty = 1). Phylogenetic inference was performed using the maximum likelihood (ML) method [43], with 500 replicates using the General Time Reversible (GTR) as the substitution model and four gamma categories and diagrams obtained by Maximum Likelihood (ML) as initial trees. The substitution model parameters employed were estimated during the search. Branch support was estimated using 500 bootstrap replicates in RAxML software.

## Results

**Fig. 1:**
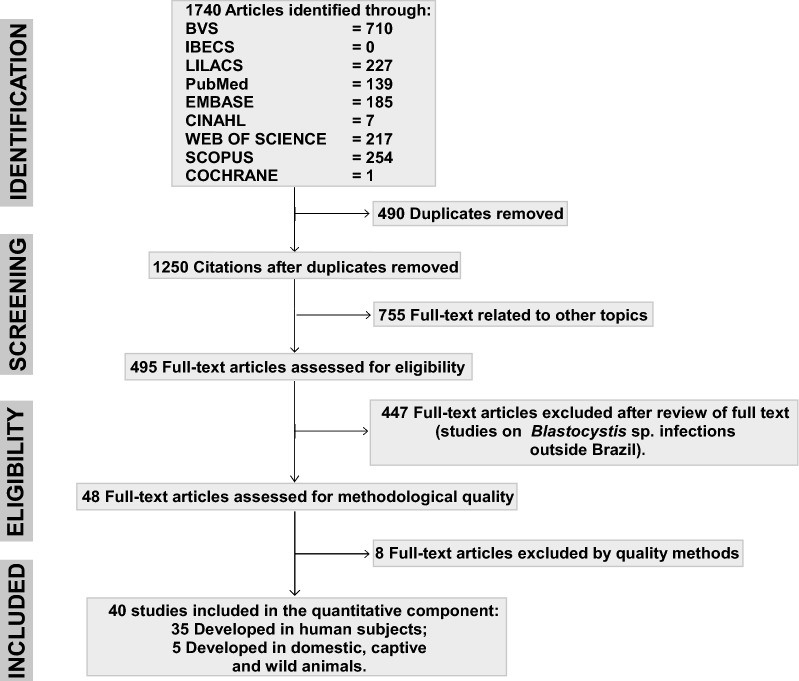
A flowchart of the steps performed in the systematic review

Our study retrieved 1740 manuscripts using the search strategies employed. After the eligibility criteria were applied (duplicate texts, articles related to other topics, text excluded based on the review or methodological quality criteria), 40 studies were retained for analysis (Table 1) [9, 36, 37, 44–80]. Of these 40 studies, 35 evaluated the prevalence of *Blastocystis* sp. in fecal samples of humans from different Brazilian states and in distinct time periods; the other five studies evaluated the prevalence of *Blastocystis* sp. infection in wild, captive and domestic animals. Ten of the 40 studies provided a molecular characterization of *Blastocystis* sp. subtypes by *SSU* rDNA partial sequencing. The results of the search strategy are shown in a PRISMA flowchart (Fig. 1). The data extracted from the final selection are provided in Additional file 2: Table S1.

**Table 1:** A summary of the included studies

No.	Reference	Total no. of tests	Prevalence (%)	City (State)	Diagnostic method
Human hosts					
1	Barbosa et al. [44]	294	55.8	Sumidouro (RJ)	C and M
2	Oliveira-Arbex et al. [45]	181	41.9	Botucatu (SP)	M
3	Seguí et al. [46]	766	28.2	Paranaguá (PR)	C and M
4	Valença-Barbosa et al. [47]	180	35.5	Duque de Caxias (RJ)	C and M
5	Faria et al. [48]	3245	2.9	Metropolitan region (RJ)	C
6	Melo et al. [49]	60	78.3	São Paulo (SP)	C and M
7	Seguí et al. [50]	217	31.8	Paranaguá (PR)	C
8	Rebolla et al. [51]	205	83.4	Sebastião da Grama (SP)	C
9	Cabrine-Santos et al. [52]	1323	17.8	Uberaba (MG)	C
10	David et al. [9]	126	53.2	Botucatu e Santa Maria da Serra (SP)	C and M
11	Santos et al. [53]	1	100	Niterói (RJ)	C and M
12	Gil et al. [54]	1338	21.2	Belo Horizonte (MG)	C
13	Gil et al. [55]	110	24.5	Sete Lagoas (MG)	C
14	Santos et al. [56]	97	13.4	Ilhéus (BA)	C
15	Amâncio et al. [57]	105	2.8	Botucatu (SP)	C
16	Branco et al. [58]	185	2.2	Campos do Jordão (SP)	C
17	Batista et al. [59]	1754	0.7	São Paulo (SP); Belo Horizonte e Uberlândia (MG); Fortaleza (CE)	C
18	Malheiros et al. [60]	382	17.3	Confresa (MT)	C and M
19	Visser et al. [61]	362	0.3	Manaus (AM)	C
20	Eymael et al. [62]	100	40.0	Novo Hamburgo (RS)	C
21	Borges et al. [63]	83	57.8	Oriximiná (PR)	C
22	Takizawa et al. [64]	343	10.7	Cascavel (PR)	C
23	Kulik et al. [65]	86	20.9	Campo Mourão (PR)	C
24	Miné et al. [66]	503	4.6	Américo Brasiliense, Gavião Peixoto, Motuca, Rincão e Araraquara (SP)	C
25	Aguiar et al. [67]	313	40.9	Sidrolândia (MS)	C
26	Alarcón et al. [68]	272	19.9	São Paulo (SP)	C
27	Carvalho-Costa et al. [69]	213	1.4	Rio de Janeiro (RJ)	C
28	Souza-Júnior et al. [70]	393	0.5	Goiânia (GO)	C
29	Nascimento et al. [71]	181	26.5	Pitanga (PR)	C
30	Amato-Neto et al. [72]	227	38.3	São Paulo (SP)	C
31	Quadros et al. [73]	200	0.5	Lages (SC)	C
32	Cimerman et al. [74]	200	0.5	São Paulo (SP)	C
33	Guimarães et al. [75]	147	32	Botucatu (SP)	C
34	Kobayashi et al. [76]	222	37.8	Holambra (SP)	C
35	Guimarães et al. [77]	173	34.7	Botucatu (SP)	C
Animal hosts					
36	Valença-Barbosa et al. [36]	89 non-human primates; 2 raccoons; 11 rodents; 26 marsupials; 1 armadillo; 57 birds; 39 pigs; 13 reptiles; 96 cockroaches	37.0; 0; 64.0; 81.0; 100; 21.0; 77.0; 69.0; 2.0. respectively	Metropolitan region (RJ)	M
37	Moura et al. [37]	78 dogs; 16 cats; 18 pigs; 28 cattle; 3 sheep	2.6; 0; 72.2; 21.4; 33.3, respectively	Uberaba (MG)	C and M
38	Marques et al. [78]	130 (bird)	2.3	Contagem, Poços de Caldas, São Gonçalo do Rio Abaixo, Betim, Belo Horizonte (MG)	C
39	Marietto-Gonçalves et al. [79]	207 (bird)	1.4	Botucatu (SP)	C
40	Mundim et al. [80]	79 (boar)	12.6	Uberlândia (MG)	C

*Abbreviations*: RJ, Rio de Janeiro; SP, São Paulo; MG, Minas Gerais; CE, Ceará; PR, Paraná; RS, Rio Grande do Sul; SC, Santa Catarina; BA, Bahia; MT, Mato Grosso; MS, Mato Grosso do Sul; GO, Goiás; AM, Amazonas; C, conventional methods, based on optical microscopy detection; M, molecular methods, based on DNA detection

Among the 35 studies utilizing human samples, 34 had high methodological quality, with a score of five. Only one study had a score of two; this study showed a risk of bias, imprecision and bias publication (small sample size). Publication bias was not assessed because currently available methods are not considered useful in studies on proportions. The five studies that evaluated the prevalence of *Blastocystis* sp. in different animal species (wild, captive and domestic animals) also showed high methodological quality, with a score of five. The I^2^ test indicated low heterogeneity among the studies. The summaries of methodological quality and bias risk and applicability for each study and among the included studies are shown in Additional file 3: Figure S1 and Additional file 4: Figure S2.

### *Blastocystis* sp. in the human population of Brazil

For the 35 studies that included human samples, 14,917 coprological tests were performed, including samples from patients from different Brazilian states. Regarding the distribution of tests performed according to the states studied, five studies were performed in the state of Rio de Janeiro, representing 26.4% of the study samples, three studies in Minas Gerais (18.6%), 13 in São Paulo (17.5%) and six in Paraná (11.2%). Only one study each was conducted in the states of Goiás, representing 4.8% of the samples included, Mato Grosso (2.6%), Amazonas (2.4%), Mato Grosso do Sul (2.1%), Santa Catarina (1.3%), Rio Grande do Sul (0.7%) and Bahia (0.7%). Finally, one study analyzed patient samples from the states of São Paulo, Minas Gerais and Ceará, which represented 11.8% of the samples included in this meta-analysis.

Of the 35 studies analyzed, only 15 classified patient samples by sex, totaling 7948 samples (51.5% female and 48.5% male). Of these, only eight reported the distribution of positive tests according to sex in 2662 samples analyzed, with 1233 (43.7%) males and 1429 (56.3%) females.

Regarding the health status of the immune system, 11,503 (81.3%) samples were from patients without a previously reported compromised immune system; the remaining 2648 (18.7%) samples were from immunocompromised patients. The types of immunosuppression reported were organ transplantation (66.2%), use of immunosuppressive drugs (14.8%), human immunodeficiency virus (HIV) carriers (11.5%) and hemodialysis (7.4%).

### Pooled prevalence of *Blastocystis* sp.

**Fig. 2:**
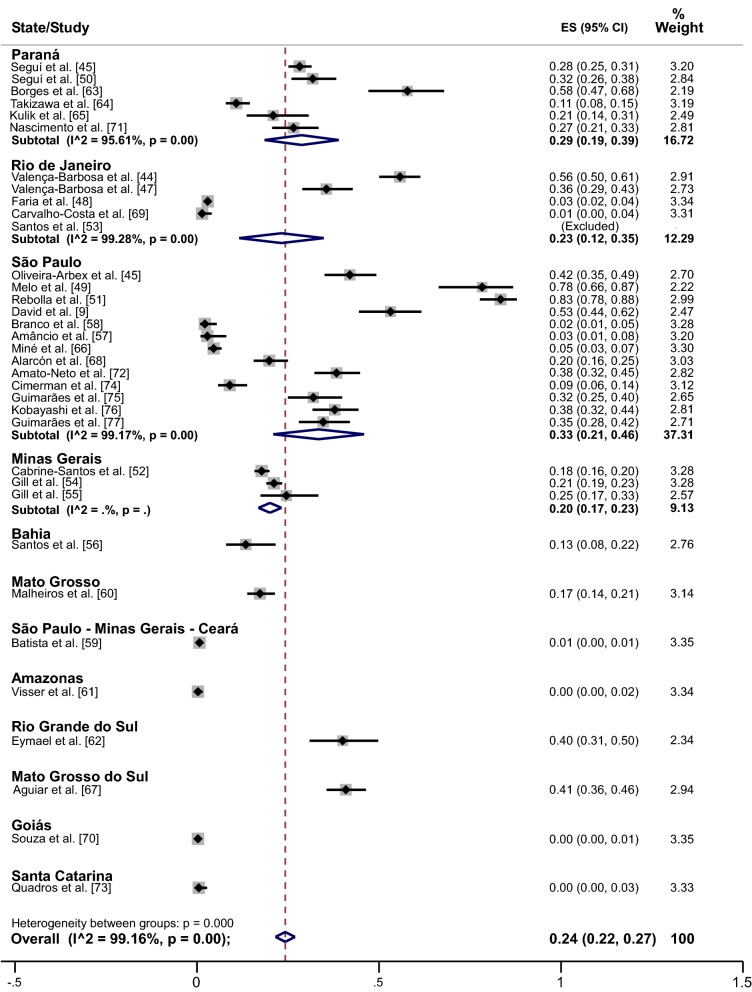
Forest plot for a random-effect meta-analysis of *Blastocystis* sp. infection in the Brazilian population

The prevalence of *Blastocystis* sp. infection reported in the studies analyzed ranged from 0.30% to 83.4%. One study included only one patient who was positive for this parasite. When the meta-analysis was performed using a random-effect model, we found an estimated pooled prevalence for *Blastocystis* sp. infection in the general Brazilian population of 24.0% (95% CI: 22.0–27.0%; weight 100%) (Fig. 2).

When the pooled prevalence was analyzed per state, we found a prevalence of 41.0% for Mato Grosso do Sul, 40.0% for Rio Grande do Sul, 33.0% for São Paulo, 29.0% for Paraná, 23.0% for Rio de Janeiro, 20.0% for Minas Gerais, 17.0% for Mato Grosso, 13.0% for Bahia, 0% for Amazonas, 0% for Goiás, and 0% for Santa Catarina. Finally, the study that analyzed samples from São Paulo, Minhas Gerais and Ceará showed a pooled prevalence of 1.0% (Fig. 2). The pooled prevalence with complete 95% CI values for each state is shown in Table 2.

**Table 2:** Distribution of the pooled prevalence of *Blastocystis* sp. infection according to state and sex

State	Overall prevalence (%)	95% CI	Weight (%)	Male	95% CI	Weight (%)	Female	95% CI	Weight (%)
Mato Grosso do Sul	41.0	36.0–46.0	29.4	36.0	29.0–43.0	13.0	47.0	39.0–55.0	12.4
Rio Grande do Sul	40.0	31.0–50.0	23.4	ns	ns	ns	ns	ns	ns
São Paulo	33.0	21.0–46.0	37.3	7	4.00–10.0	25.0	6,00	4.00–9.00	24.6
Paraná	29.0	19.0–39.0	16.7	28.0	24.0–33.0	25.0	27.0	23.0–31.0	25.4
Rio de Janeiro	23.0	12.0–35.0	12.3	63.0	55.0–71.0	13.0	48.0	40.0–56.0	12.4
Minas Gerais	20.0	17.0–23.0	9.13	ns	ns	ns	ns	ns	ns
Mato Grosso	17.0	14.0–21.0	3.14	20.0	15.0–27.0	13.0	15.0	11.0–20.0	12.7
Bahia	13.0	8.00–22.0	2.76	19.0	11.0–31.0	11.0	7.00	2.00–18.0	12.5
Amazonas	0	0.00–2.00	3.34	ns	ns	ns	ns	ns	ns
Goiás	0	0.00–1.00	3.35	ns	ns	ns	ns	ns	ns
Santa Catarina	0	0.00–3.00	3.33	ns	ns	ns	ns	ns	ns
São Paulo, Minhas Gerais and Ceará^a^	1.00	0.00–1.00	3.35	ns	ns	ns	ns	ns	ns

^a^A single study included from these states

*Abbreviations*: ns, not specified; 95% CI, 95% confidence interval

The pooled prevalence calculated for the 1233 male samples was 31.0% (95% CI: 17.0–45.0%; weight 100%); the state with the highest prevalence was Rio de Janeiro, (63.0%), followed by Mato Grosso do Sul (36.0%); Paraná (28.0%); Mato Grosso (20.0%); Bahia (19.0%;) and São Paulo (7.0%). In turn, the pooled prevalence calculated for the 1429 female samples was 28.0% (95% CI: 16.0–41.0%; weight 100%); the state with the highest prevalence was Rio de Janeiro (48.0%), followed by Mato Grosso do Sul (47.0%), Paraná (27.0%), Mato Grosso (15.0%), Bahia (7.0%) and São Paulo (6.0%). The pooled prevalence with complete 95% CI values for each state by sex is shown in Table 2.

Among patients without a compromised immune system, the pooled prevalence was 29.0% (95% CI: 24.0–33.0%; weight 100%), whereas the pooled prevalence for immunosuppressed patients was 5.0% (95% CI: 3.0–7.0%; weight 100%). The cause of immunosuppression most prevalent with *Blastocystis* sp. infection was hemodialysis, at 23.0%, followed by HIV infection at 5.0%, organ transplant at 1.0% and immunosuppressive drug use at 1.0%. The pooled prevalence with complete 95% CI values for each type of immunosuppression is shown in Table 3.

**Table 3:** Distribution of the pooled prevalence of *Blastocystis* sp. infection according to the type of immunosuppression

Type of immunosuppression	Overall prevalence (%)	95% CI	Weight (%)
Hemodialysis	23.0	17.0–29.0	12.4
HIV infection	5.0	5.00–8.00	33.6
Organ transplant	1.0	0.00–1.00	27.2
Immunosuppressive drugs	1.0	0.00–2.00	26.8

*Abbreviations*: 95% CI, 95% confidence interval

### *Blastocystis* sp. in animals from Brazil

In the five studies that analyzed the prevalence of *Blastocystis* sp. in animals in Brazil, 892 coprological tests were performed on different species of mammals, birds and reptiles. Regarding the classification of these animals, 65.0% were birds, 20.0% were mammals, and 15.0% were reptiles. Regarding classification according to their direct interaction with humans, 42.3% were wild, 37.4% were domestic, and 20.3% were in captivity.

**Fig. 3:**
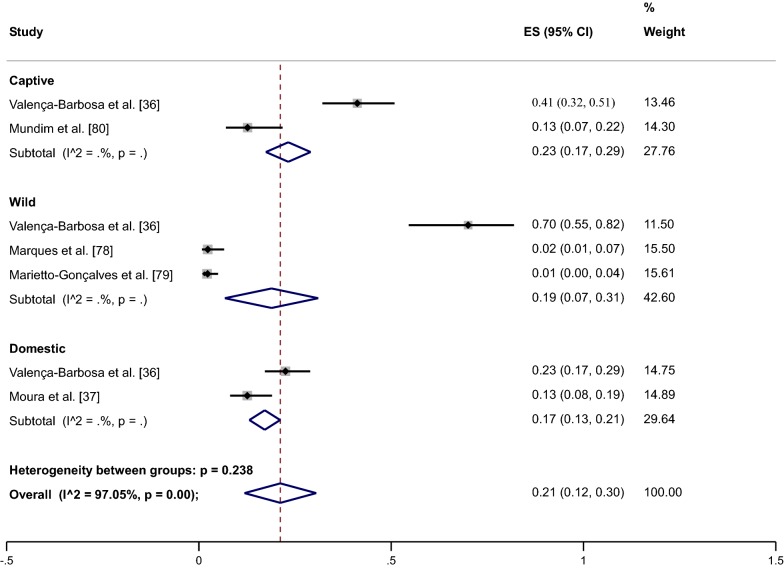
Forest plot for a random-effect meta-analysis of *Blastocystis* sp. infection in different taxonomic classes of animals in Brazil, according to the type of interaction with humans

After analyzing the infection by *Blastocystis* sp. in animals in Brazil of different orders and with different types of interaction with humans, a pooled prevalence of 21.0% (95% CI: 12.0–37.0%; weight 100%) was observed. The prevalence of *Blastocystis* sp. according to taxonomic class showed the highest percentage of infection among mammals, at 39.0%, followed by birds (18.0%) and reptiles (3.0%). Captive animals represented 23.0%, followed by wild animals at 19.0% and domestic animals at 17.0% (Fig. 3).

Among the mammals in captivity, non-human primates were the most studied, with high prevalence rates among *Macaca mulata* (60.0%) and *Macaca fascicularis* (35.0%). *Didelphis aurita* was the wild mammal with the highest prevalence (76.0%). Finally, among domestic mammals, *Sus scrofa* represented 24.0% and *Bos taurus* 21.0%. Notably, the only animals considered pets included in the analyzed studies, *Canis lupus familiaris* and *Felis catus*, had pooled prevalences of 3.0% and 0%, respectively.

Among birds, only species with domestic and wild interactions were studied. *Anser anser* showed a prevalence of 70.0% and *Anas platyrhynchos domesticus* a prevalence of 23.0%. Finally, wild birds positive for *Blastocystis* sp. were *Penelope obscura*, with a prevalence of 4.0%, and *Oryzoborus angolensis*, with a prevalence of 6.0%. The *Chelonoidis* sp., the only reptile species with captive interaction, showed a prevalence of 69.9% (95% CI: 42.0–87.0%; weight 5.10%). Finally, the cockroach *Periplaneta americana* showed a prevalence of 2.0% (95% CI: 1.0–7.0%; weight 7.54%). The pooled prevalence with complete 95% CI values for each taxonomic class and species of animal are shown in Table 4.

**Table 4:** Distribution of the pooled prevalence of *Blastocystis* sp. according to taxonomic class and species

Taxonomic class	Overall prevalence (%)	95% CI	Weight (%)
Mammals	39.0	21.0–56.0	47.31
*Macaca mulatta*	60.0	42.0–75.0	6.13
*Macaca fascicularis*	35.0	18.0–57.0	5.67
*Didelphis aurita*	76.0	57.0–89.0	6.23
*Sus scrofa*	24.0	16.0–33.0	7.19
*Bos taurus*	21.0	10.0–40.0	6.43
*Canis lupus familiaris*	3.0	1.0–9.0	7.52
*Felis catus*	0	0.0–5.0	15.66
Birds	18.0	10.0–27.0	39.31
*Anser anser*	70.0	62.0–87.0	6.68
*Anas platyrhynchos domesticus*	23.0	13.0–37.0	6.78
*Penelope obscura*	4.0	1.0–18.0	6.78
*Oryzoborus angolensis*	6.0	2.0–19.0	7.25
Reptile	3.0	0.0–0.06	13.38
*Chelonoidis* sp.	69.9	42.0–87.0	5.10
Interaction with humans			
Captive	23.0	17.0–29.0	27.76
Wild	19.0	7.0–31.0	42.60
Domestic	17.0	13.0–21.0	29.64

*Abbreviations*: 95% CI, 95% confidence interval

### Diversity of *Blastocystis* sp. subtypes in different host species in Brazil

*SSU* rDNA was used in 10 studies to identify *Blastocystis* sp. subtypes in samples from different host species. Of these studies, eight were performed in humans and two in animals of different classes. In total, 473 samples from humans and 118 from animals were studied.

**Fig. 4:**
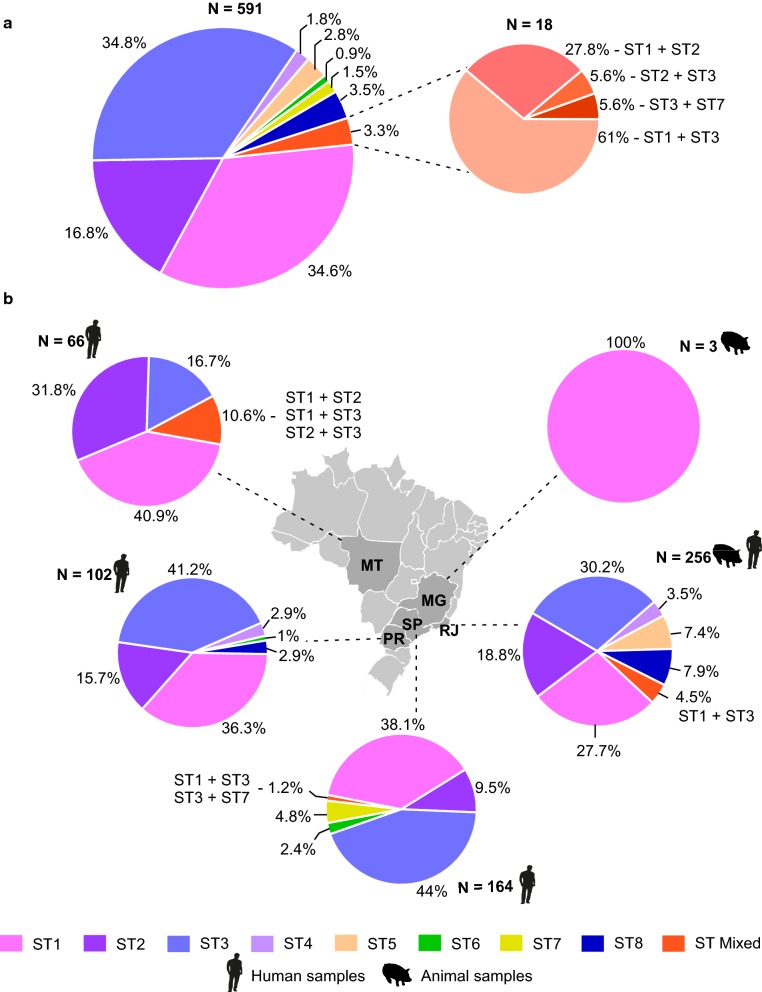
Geographical distribution of *Blastocystis* subtypes detected in Brazil. **a** Subtypes detected in 473 human and 118 animal samples. **b** Distribution of subtypes in Brazilian states. In the state of Rio de Janeiro, all STs found in human samples were also found in animal samples. *Abbreviations*: MT, Mato Grosso; SP, São Paulo; RJ, Rio de Janeiro; PR, Paraná; MG, Minas Gerais

The *Blastocystis* subtypes identified in the different hosts were ST1 to ST8, with the most prevalent being ST1, ST2 and ST3, with an overall percentage of infection of 86.2% (Fig. 4).

Although ST1 was more prevalent in humans (36.8%), it was also detected in domestic and wild mammals and birds (19.2%). In human hosts, ST1 was detected in the states of Mato Grosso, São Paulo, Paraná and Rio de Janeiro. In pigs, ST1 was identified in the state of Minas Gerais; in Rio de Janeiro, it was detected in non-human primates, marsupials, wild boars and birds.

ST2 and ST3 were detected in human samples from the states of Mato Grosso, São Paulo, Paraná and Rio de Janeiro. Both were found in samples from non-human primates in the state of Rio de Janeiro, and ST3 was also detected in a rodent in the same state (Fig. 4).

ST4 was the least prevalent (1.8%) and was found in human hosts in the states of Paraná and Rio de Janeiro. This subtype was also found in wild boar and cockroach samples and was the only subtype detected in insects in Brazil. ST5 was found only in rooster and wild boar samples in the state of Rio de Janeiro. ST6 and ST7 were identified only in human hosts in the states of São Paulo and Paraná. ST8 was identified in humans, non-human primates, marsupials, armadillo and wild boars in the states of Rio de Janeiro and Paraná (Fig. 4).

### Phylogenetic analysis of *Blastocystis* sp. subtypes found in Brazil

To understand phylogenetic relationships between the subtypes and their interactions with their hosts, a phylogenetic analysis was performed using the ML estimation method, and 255 sequences of ST1-ST5, ST8, ST11, ST12 and ST14 were included. The accession numbers for the GenBank sequences are provided in Additional file 5: Table S2.

Of the subtypes found in Brazil (ST1 to ST8), only ST6 and ST7 were not included due to a lack of sequences compatible with the *SSU* rDNA gene fragment used to perform the alignment. The alignment used to perform the phylogenetic inference is provided in Additional file 6: Text S2.

**Fig. 5:**
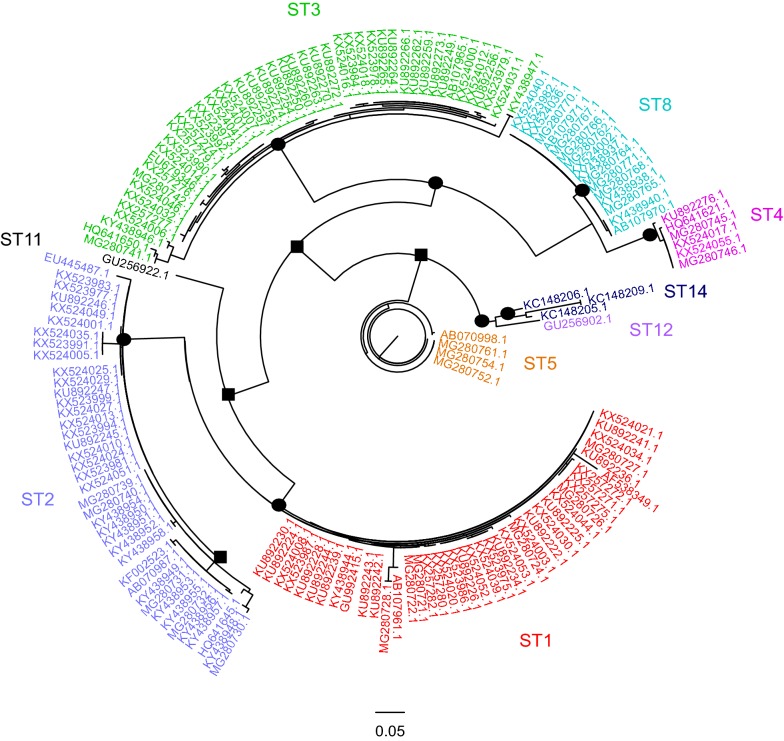
A dendrogram inferred by Maximum Likelihood analysis using 255 sequences of the *SSU* rRNA gene fragment (365 characters, see alignment in Additional file 6: Text S2). Bootstrap node support values ≥ 0.95 are shown as circles at the nodes, and bootstrap node support values of 0.75–0.94 are shown as squares at the nodes

The unrooted tree presents nine clades that correspond exactly to each ST included in the analysis (Fig. 5). Each subtype was strongly supported by a high bootstrap value. Furthermore, the results showed a relationship between clades ST1 and ST2, among clades ST5, ST12 and ST14, and between clades ST4 and ST8. Clades ST1, ST2 and ST3 exhibited greater genetic diversity because they have at least two possible genotypes in each ST. With the exception of ST11, of which only one sequence was included, the other clades included in the inference were shown to be genetically homogeneous (Fig. 5).

## Discussion

A pooled prevalence for *Blastocystis* sp. infection in the general Brazilian population of 24% was found in this meta-analysis. Of the five Brazilian regions, the highest amount of published data was for the South, South-East and Center-West regions. A total of 32 articles representing approximately 85.1% of the samples analyzed in this meta-analysis were included in these regions. Regarding the North and North-East regions, only one study per region was included, representing 3.1% of the samples analyzed. Notably, one study used samples from the North-East and South-East regions (São Paulo, Minas Gerais and Ceará), but the results were not segregated by state. This study included 11.8% of the samples analyzed. Regarding sex, both showed similar prevalence rates: 31% for men and 28% for women.

There are contrasting realities within the states that compose each region. In the Center-West, we observed high prevalence rates in the states of Mato Grosso do Sul (41.0%) and Mato Grosso (17.0%), yet the pooled prevalence in Goiás was 0%. The same phenomenon was observed in the South and South-East regions, where states such as Rio Grande do Sul (40.0%), São Paulo (33.0%), Paraná (29.0%) and Minas Gerais (20.0%) had high prevalence rates, but the same was not true for Santa Catarina (0%). Importantly, a significant number of samples was analyzed in these studies conducted in both Goiás and Santa Catarina, decreasing the probability of sampling error. However, in the few studies conducted in the states of Amazonas and Bahia, which are the only representatives of the North and North-East regions, the calculated prevalence rates were 0% and 13.0%, respectively. Regardless, further studies should be conducted to corroborate these rates in these regions.

Brazil is a country with many regional differences in climatic conditions and socioeconomic development. According to data from the Ministry of Cities (Sistema Nacional de Informações sobre Saneamento (National Sanitation Information System 2014, SNIS) [81], only 39% of the municipalities collect and treat 100% of their sewage. The lack of an appropriate waste collection and sewage treatment system can contribute to the dissemination of neglected diseases, including those caused by *Blastocystis* sp.

The prevalence of *Blastocystis* sp. in immunocompromised patients was 5.0% (95% CI: 3.0–7.0%; weight 100%). Patients under hemodialysis treatment were the most susceptible to infection by this parasite, at 23.0% (95% CI: 17.0–29.0%; weight 12.36%). HIV patients ranked second, with a prevalence of 5.0% (95% CI: 5.0–8.0%; weight 33.61%).

Some studies indicate that this parasite often causes opportunistic infection in immunosuppressed patients [65], specifically diarrhea that is usually accompanied by weight loss, vomiting, malabsorption syndrome and, in some cases, fever and abdominal pain [82]. Other studies have reported that *Blastocystis* sp. is responsible for clinically relevant infections in patients undergoing hemodialysis and kidney transplantation; the prevalence rates in these studies vary between 15–25% and 20–35%, respectively [22, 23, 25, 26]. Our results showed low prevalence rates in patients with organ transplants. As patients undergoing hemodialysis are candidates for kidney transplantation, planning strategies for the prevention of parasitic infections and appropriate interventions are necessary to improve the quality of life of these patients.

Furthermore, our results revealed a wide diversity of hosts, including animals of various orders (mammals, birds and reptiles) and even insects (cockroaches) capable of harboring and distributing *Blastocystis* sp. Among these animals, mammals showed the highest prevalence rates, at 39.0% (95% CI: 21.0–56.0%; weight 47.31%), followed by birds at 18.0% (95% CI: 10.0–27.0%; weight 39.31%). In regard to interaction with humans, the highest prevalence rates were observed for captive animals, which are not easily accessible to the population. Wild animals and domestic animals had the second highest prevalence rates. Although domestic animals had lower prevalence rates, a prevalence of 17.0% (95% CI: 13.0–21.0%; weight 29.64%), which is still significant, was observed. When domestic animals were analyzed, we found them to be animals related to livestock production. Among these animals, the wild boar (*Sus scrofa*) and the ox (*Bos taurus*) among mammals and the Greylag goose (*Anser anser*) and the domestic duck (*Anas platyrhynchos domesticus*) among birds had the highest prevalence rates. Notably, the prevalence rate among animals considered to be pets (cat and dog) was low. Thus, our results point to rearing livestock as a possible risk factor for *Blastocystis* sp., and control measures against intestinal parasites should be reinforced to minimize the transmission of these parasites through the feces of these animals.

Our phylogenetic inference revealed a relationship between ST1 and ST2 as well as between ST8 and ST4. Such a relationship was also proposed in a recent study that analyzed *SSU* rDNA sequences in fecal samples from animals of various orders [36]. After analyzing sequences from several STs in fecal samples from humans and other mammals, birds, reptiles and cockroaches, we observed a considerable overlap between different hosts and the *Blastocystis* subtypes. Therefore, no specific host-ST relationship could be detected. Because the ST2 sequences analyzed were from humans and non-human captive primates, it seems reasonable to assume the possibility of zoonotic transmission where these animals are kept. Other possible interactions may occur through contacts with domestic animals, especially on farms (ST1 and ST4).

The correlation between pathogenicity and *Blastocystis* sp. STs has been the subject of several studies, showing that not all strains of a subtype are pathogenic and suggesting that subtype is not the only factor related to pathogenicity [15, 16]. Indeed, our phylogenetic analysis suggests the possibility that more than one strain of ST1, ST2 and ST3 may result in different clinical symptoms in infected patients.

There are some limitations to our study. First, in the studies conducted in humans, some authors did not segregate the results of positive samples by sex, which decreased the number of samples available to evaluate prevalence for this variable. Secondly, the samples per state were not segregated in one study [59], but this may contribute to a better calculation of the prevalence rates in the states involved. Thirdly, our results only reflect the reality in the South, South-East and Center-West regions, which have higher scientific production. Fourthly, the lack of partial *SSU* rDNA sequences available for ST6, ST7, ST9, ST10, ST13, ST15, ST16 and ST17 led to the need to exclude these STs, limiting the phylogenetic analysis. Finally, in meta-analyses, it is recommended that publication bias be assessed through statistical methods. However, currently available methods such as funnel plots and Eggerʼs regression test are not considered useful in studies on proportions [83]. Additionally, the statistical power of these tests is affected by the presence of high heterogeneity and the limited number of studies [84]. Accordingly, publication bias was not measured.

## Conclusions

This study revealed a high prevalence (24%) of *Blastocystis* sp. in the Brazilian population, a value that was influenced by the most studied regions (South, South-East and Center-West), where prevalence rates of up to 40% were found. Among animals, mammals and birds exhibited the highest prevalence rates, and domestic animals used as livestock are possibly most related to parasite transmission. Eco-epidemiological studies of *Blastocystis* sp. are very important due to the possible interactions of host animals with humans. Therefore, control measures against intestinal parasites should be reinforced to prevent the transmission of these parasites, principally in zoos and on farms. Finally, in patients with any type of immunosuppression, routine screening of opportunistic intestinal protozoa should be performed, and early treatment should be administered.

## Supplementary information

**Additional file 1: Text S1.** The search strategy.

**Additional file 2: Table S1.** PRISMA checklist of items to include when reporting a meta-analysis.

**Additional file 3: Figure S1.** The summary of methodological quality and bias risk and applicability for each study.

**Additional file 4: Figure S2.** The summary of methodological quality and bias risk and applicability across the included studies.

**Additional file 5: Table S2.** The accession numbers for GenBank sequences.

**Additional file 6: Text S2.** The alignment used to perform phylogenetic inference.

## Data Availability

All data are presented in the manuscript and its additional files.

## Abbreviations

ST: subtype
RNA: ribonucleic acid
SSU: small subunit
rDNA: ribosomal deoxyribonucleic acid
NMASTs: non-mammal and avian host STs
PROSPERO: Prospective Register of Systematic Reviews
MeSH: Medical Subject Headings
IBECS: The Spanish Bibliographic Index of the Health Sciences
LILACS: The Latin American and Caribbean Health Sciences Literature
Medline: The United States National Library of Medicine Bibliographic Database
EMBASE: The Elsevier database
The Cochrane Library and NICE: National Institute for Health and Clinical Excellence
GRADE: grading of recommendations assessment development and evaluation
RevMan: review manager
I^2^: the Higgins test
ML: maximum likelihood
GTR: general time reversible
PRISMA: preferred reporting items for systematic reviews and meta-analyses
HIV: human immunodeficiency virus
CI: confidence interval
MT: Mato Grosso
SP: São Paulo
RJ: Rio de Janeiro
PR: Paraná
MG: Minas Gerais

## References

[1] Stensvold CR, Lewis HC, Hammerum AM, Porsbo LJ, Nielsen SS, Olsen KE (2009). *Blastocystis*: unraveling potential risk factors, and clinical significance of a common but neglected parasite. Epidemiol Infect..

[2] Tan KS, Singh M, Yap EH (2010). Current views on the clinical relevance of *Blastocystis* spp. Curr Infect Dis Rep..

[3] Stenzel D, Boreham P (1996). *Blastocystis hominis* revisited. Clin Microbiol Rev..

[4] Li LH, Zhang XP, Lv S, Zhang L, Yoshikawa H, Wu Z (2007). Cross-sectional surveys and subtype classification of human *Blastocystis* isolates from four epidemiological settings in China. Parasitol Res..

[5] Leelayoova S, Siripattanapipong S, Thathaisong U, Naaglor T, Taamasri P, Piyaraj P (2008). Drinking water: a possible source of *Blastocystis* spp. subtype 1 infection in schoolchildren of a rural community in central Thailand. Am J Trop Med Hyg..

[6] Eroglu F, Koltas IS (2010). Evaluation of the transmission mode of *B. hominis* by using PCR method. Parasitol Res..

[7] WHO. Guidelines for drinking-water quality. 3rd ed. Geneva: World Health Organization; 2008. https://www.who.int/water_sanitation_health/publications/gdwq3/en/. Accessed 30 Dec 2019.

[8] Souppart L, Sanciu G, Cian A, Wawrzyniak I, Delbac F, Capron M (2009). Molecular epidemiology of human *Blastocystis* isolates in France. Parasitol Res..

[9] David EB, Guimaraes S, Oliveira AP, Oliveira-Sequeira TCG, Bittencourt GN, Nardi ARM (2015). Molecular characterization of intestinal protozoa in two poor communities in the State of Sao Paulo, Brazil. Parasites Vectors..

[10] Balint A, Doczi I, Bereczki L, Gyulai R, Szucs M, Farkas K (2014). Do not forget the stool examination!-cutaneous and gastrointestinal manifestations of *Blastocystis* sp. infection. Parasitol Res..

[11] Ramirez JD, Sanchez LV, Bautista DC, Corredor AF, Florez AC, Stensvold CR (2014). *Blastocystis* subtypes detected in humans and animals from Colombia. Infect Genet Evol..

[12] Abu-Madi M, Aly M, Behnke JM, Clark CG, Balkhy H (2015). The distribution of *Blastocystis* subtypes in isolates from Qatar. Parasites Vectors..

[13] Pandey PK, Verma P, Marathe N, Shetty S, Bavdekar A, Patole MS (2015). Prevalence and subtype analysis of *Blastocystis* in healthy Indian individuals. Infect Genet Evol..

[14] Requena-Certad I, Devera R, Agreda Y, Cordova Y, Castillo H, Velasquez V (1999). Infección por *Blastocystis hominis* en pacientes pediátricos hospitalizados. Rev Biomed..

[15] Roberts T, Stark D, Harkness J, Ellis J (2014). Update on the pathogenic potential and treatment options for *Blastocystis* sp. Gut Pathog..

[16] Wawrzyniak I, Poirier P, Viscogliosi E, Dionigia M, Texier C, Delbac F (2013). *Blastocystis*, an unrecognized parasite: an overview of pathogenesis and diagnosis. Ther Adv Infect Dis..

[17] Mirza H, Tan KSW (2009). *Blastocystis* exhibits inter- and intra-subtype variation in cysteine protease activity. Parasitol Res..

[18] Puthia MK, Sio SW, Lu J, Tan KS (2006). *Blastocystis ratti* induces contact-independent apoptosis, F-actin rearrangement, and barrier function disruption in IEC-6 cells. Infect Immun..

[19] Stensvold CR, Christiansen DB, Olsen KE, Nielsen HV (2011). *Blastocystis* sp. Subtype 4 is common in Danish *Blastocystis*-positive patients presenting with acute diarrhea. Am J Trop Med Hyg..

[20] Ajjampur SS, Tan KS (2016). Pathogenic mechanisms in *Blastocystis* spp.—interpreting results from *in vitro* and *in vivo* studies. Parasitol Int..

[21] Dogruman-Al F, Kustimur S, Yoshikawa H, Tuncer C, Simsek Z, Tanyuksel M (2009). *Blastocystis* subtypes in irritable bowel syndrome and inflammatory bowel disease in Ankara, Turkey. Mem Inst Oswaldo Cruz..

[22] Ali MS, Mahmoud LA, Abaza BE, Ramadan MA (2000). Intestinal spore-forming protozoa among patients suffering from renal failure. J Egypt Soc Parasitol..

[23] Chieffi PP, Sens YAS, Paschoalotti MA, Miorin LA, Silva HGC, Jabur P (1998). Infection by *Cryptosporidium parvum* in renal patients submitted to renal transplant or hemodialysis. Rev Soc Bras Med Trop..

[24] Minz M, Udgiri NK, Heer MK, Kashyap R, Malla N (2004). Cryptosporidiasis in live related renal transplant recipients: a single center experience. Transplantation..

[25] Sela S, Shurtz-Swirski R, Cohen-Mazor M, Mazor R, Chezar J, Shapiro G (2005). Primed peripheral polymorphonuclear leukocyte: a culprit underlying chronic low-grade inflammation and systemic oxidative stress in chronic kidney disease. J Am Soc Nephrol..

[26] Turkcapar N, Kutlay S, Nergizoglu G, Atli T, Duman N (2002). Prevalence of *Cryptosporidium* infection in hemodialysis patients. Nephron..

[27] Alfellani MA, Taner-Mulla D, Jacob AS, Imeede CA, Yoshikawa H, Stensvold CR (2013). Genetic diversity of *Blastocystis* in livestock and zoo animals. Protist..

[28] Stensvold CR, Alfellani M, Clark CG (2012). Levels of genetic diversity vary dramatically between *Blastocystis* subtypes. Infect Genet Evol..

[29] Stensvold CR, Clark CG (2016). Current status of *Blastocystis*: a personal view. Parasitol Int..

[30] Yoshikawa H, Wu Z, Pandey K, Pandey BD, Sherchand JB, Yanagi T (2009). Molecular characterization of *Blastocystis* isolates from children and rhesus monkeys in Kathmandu, Nepal. Vet Parasitol..

[31] Parkar U, Traub RJ, Vitali S, Elliot A, Levecke B, Robertson I (2010). Molecular characterization of *Blastocystis* isolates from zoo animals and their animal-keepers. Vet Parasitol..

[32] Yan Y, Su S, Ye J, Lai X, Lai R, Liao H (2007). *Blastocystis* sp. subtype 5: a possibly zoonotic genotype. Parasitol Res..

[33] Noel C, Dufernez F, Gerbod D, Edgcomb VP, Delgado-Viscogliosi P, Ho LC (2005). Molecular phylogenies of *Blastocystis* isolates from different hosts: implications for genetic diversity, identification of species, and zoonosis. J Clin Microbiol..

[34] Clark CG, Van der Giezen M, Alfellani MA, Stensvold CR (2013). Recent developments in *Blastocystis* research. Adv Parasitol..

[35] Ramirez JD, Sanchez A, Hernandez C, Florez C, Bernal MC, Giraldo JC (2016). Geographic distribution of human *Blastocystis* subtypes in South America. Infect Genet Evol..

[36] Valença-Barbosa C, Bomfim TCB, Teixeira BR, Gentile R, Costa Neto SFC, Magalhães BSN (2019). Molecular epidemiology of *Blastocystis* isolated from animals in the state of Rio de Janeiro, Brazil. PLoS ONE..

[37] Moura RGF, Oliveira-Silva MB, Pedrosa AL, Nascentes GAN, Cabrine-Santos M (2018). Occurrence of *Blastocystis* spp. in domestic animals in Triângulo Mineiro área of Brazil. Rev Soc Bras Med Trop..

[38] Stensvold CR (2013). Comparison of sequencing (barcode region) and sequence-tagged-site PCR for *Blastocystis* subtyping. J Clin Microbiol..

[39] Cian A, Safadi DE, Osman M, Moriniere R, Gantois N, Benamrouz-Vanneste S (2017). Molecular epidemiology of *Blastocystis* sp. in various animal groups from two French zoos and evaluation of potential zoonotic risk. PLoS ONE.

[40] Yoshikawa H, Koyama Y, Tsuchiya E, Takami K (2016). *Blastocystis* phylogeny among various isolates from humans to insects. Parasitol Int..

[41] Higgins JPT, Green S, editors. Cochrane handbook for systematic reviews of interventions. Version 5.1.0. London: The Cochrane Collaboration; 2011. http://handbook-5-1.cochrane.org/. Accessed 20 Mar 2019.

[42] Thompson JD, Gibson TJ, Plewniak F, Jeanmougin F, Higgins DG (1997). The CLUSTAL_X windows interface: flexible strategies for multiple sequence alignment aided by quality analysis tools. Nucleic Acids Res..

[43] Stamatakis A (2006). RAxML-VI-HPC: maximum likelihood-based phylogenetic analyses with thousands of taxa and mixed models. Bioinformatics..

[44] Barbosa CV, Barreto MM, Andrade RJ, Sodré F, D’Avila-Levy CM, Peralta JM (2018). Intestinal parasite infections in a rural community of Rio de Janeiro (Brazil): prevalence and genetic diversity of *Blastocystis* subtypes. PLoS ONE.

[45] Oliveira-Arbex AP, David ÉB, Guimarães S (2018). *Blastocystis* genetic diversity among children of low-income daycare center in southeastern Brazil. Infect Genet Evol..

[46] Seguí R, Muñoz-Antoli C, Klisiowicz DR, Oishi CY, Köster PC, Lucio A (2018). Prevalence of intestinal parasites, with emphasis on the molecular epidemiology of *Giardia duodenalis* and *Blastocystis* sp., in the Paranaguá Bay, Brazil: a community survey. Parasites Vectors..

[47] Valença-Barbosa C, Batista RJ, Igreja RP, Levy CMD, Macedo HW, Santos HLC (2017). Distribution of *Blastocystis* subtypes isolated from humans from an urban community in Rio de Janeiro, Brazil. Parasites Vectors..

[48] Faria CP, Zanini GM, Dias GS, Silva S, Freitas MB, Almendra R (2017). Geospatial distribution of intestinal parasitic infections in Rio de Janeiro (Brazil) and its association with social determinants. PLoS Negl Trop Dis.

[49] Melo GB, Paula FM, Malta FM, Maruta CW, Criado P, Castilho VLP (2017). Identification of *Blastocystis* subtypes in clinical stool samples from Sao Paulo city, Brazil. Parasitol Open..

[50] Seguí R, Klisiowicz D, Oishi CY, Toledo R, Esteban JG, Muñoz-Antoli C (2017). Intestinal symptoms and *Blastocystis* load in schoolchildren of Paranaguá Bay, Paraná, Brazil. Rev Inst Med Trop S Paulo..

[51] Rebolla MF, Silva EM, Gomes JF, Falcao AX, Rebolla MV, Franco RM (2016). High prevalence of *Blastocystis* spp. infection in children and staff members attending public urban schools in Sao Paulo state, Brazil. Rev Inst Med Trop S Paulo..

[52] Cabrine-Santos M, Cintra Edo N, do Carmo RA, Nascentes GA, Pedrosa AL, Correia D (2015). Occurrence of *Blastocystis* spp. in Uberaba, Minas Gerais, Brazil. Rev Inst Med Trop S Paulo..

[53] Santos Helena Lúcia, Sodré Fernando, de Macedo Heloisa (2014). Blastocystis sp. in splenic cysts: causative agent or accidental association? A unique case report. Parasites & Vectors.

[54] Gil FF, Busatti HG, Cruz VL, Santos JF, Gomes MA (2013). High prevalence of enteroparasitosis in urban slums of Belo Horizonte—Brazil. Presence of enteroparasites as a risk factor in the family group. Pathog Glob Health..

[55] Gil FF, Barros MJ, Macedo NA, E. Júnior CG, Redoan R, Busatti H (2013). Prevalence of intestinal parasitism and associated symptomatology among hemodialysis patients. Rev Inst Med Trop S Paulo..

[56] Santos HLC, Martins LAF, Peralta RHS, Peralta JM, Macedo HW (2014). Frequency of amoebiasis and other intestinal parasitoses in a settlement in Ilhéus city, state of Bahia, Brazil. Rev Soc Bras Med Trop..

[57] Amâncio FAM, Pascotto VM, Souza LR, Calvi SA, Pereira PCM (2012). Intestinal parasitic infections in HIV/AIDS patients: epidemiological, nutritional and immunological aspects. J Venom Anim Toxins incl Trop Dis..

[58] Branco N, Leal DAG, Franco RMB (2012). A parasitological survey of natural water springs and inhabitants of a tourist city in southeastern Brazil. Vector Borne Zoonotic Dis..

[59] Batista MV, Pierrotti LC, Abdala E, Clemente WT, Girão ES, Rosa DRT (2011). Endemic and opportunistic infections in Brazilian solid organ transplant recipients. Trop Med Int Health..

[60] Malheiros AF, Stensvold CR, Clark CG, Braga GB, Shaw JJ (2011). Short report: molecular characterization of *Blastocystis* obtained from members of the indigenous Tapirape ethnic group from the Brazilian Amazon region, Brazil. Am J Trop Med Hyg..

[61] Visser S, Giatti LL, Carvalho RAC, Guerreiro KCJ (2011). Estudo da associação entre fatores socioambientais e prevalência de parasitose intestinal em área periférica da cidade de Manaus (AM, Brasil). Cien Saude Colet..

[62] Eymael D, Schuh GM, Tavares RG (2010). Padronização do diagnóstico de *Blastocystis hominis* por diferentes técnicas de coloração. Rev Soc Bras Med Trop..

[63] Borges JD, Alarcón RS, Amato Neto V, Gakiya E (2009). Intestinal parasitosis in Indians of the Mapuera community (Oriximiná, State of Pará, Brazil): high prevalence of *Blastocystis hominis* and finding of *Cryptosporidium* sp. and *Cyclospora cayetanensis*. Rev Soc Bras Med Trop..

[64] Takizawa MGMH, Falavigna DLM, Gomes ML (2009). Enteroparasitosis and their ethnographic relationship to food handlers in a tourist and economic center in Paraná, Southern Brazil. Rev Inst Med Trop S Paulo..

[65] Kulik RA, Falavigna DL, Nishi L, Araujo SM (2008). *Blastocystis* sp. and other intestinal parasites in hemodialysis patients. Braz J Infect Dis..

[66] Miné JC, Rosa JA (2008). Frequency of *Blastocystis hominis* and other intestinal parasites in stool samples examined at the Parasitology Laboratory of the School of Pharmaceutical Sciences at the São Paulo State University, Araraquara. Rev Soc Bras Med Trop..

[67] Aguiar JIA, Gonçalves AQ, Sodré FC, Pereira SR, Bóia MN, Lemos ERS (2007). Intestinal protozoa and helminths among Terena Indians in the state of Mato Grosso do Sul: high prevalence of *Blastocystis hominis*. Rev Soc Bras Med Trop..

[68] Alarcón RSR, Amato Neto V, Gakiya E, Bezerra RC (2007). Observações *sobre Blastocystis hominis* e *Cyclospora cayetanensis* em exames parasitológicos efetuados rotineiramente. Rev Soc Bras Med Trop..

[69] Carvalho-Costa FA, Gonçalves AQ, Lassance SL, Albuquerque CP, Leite JPG, Bóia MN (2007). Detection of *Cryptosporidium* spp. and other intestinal parasites in children with acute diarrhea and severe dehydration in Rio de Janeiro. Rev Soc Bras Med Trop..

[70] Souza-Júnior ES, Garcia-Zapata MTA (2006). Diagnóstico laboratorial de enteroparasitoses oportunistas, com ênfase nas microsporidioses humanas, em Goiânia-GO. Rev Soc Bras Med Trop..

[71] Nascimento SA, Moitinho MLR (2005). *Blastocystis hominis* and other intestinal parasites in a community of Pitanga city, Paraná State, Brazil. Rev Soc Bras Med Trop..

[72] Amato-Neto V, Alarcón RSR, Gakiya E, Ferreira CS, Bezerra RC, Santos AG (2004). Elevada porcentagem de blastocistose em escolares de São Paulo, SP. Rev Soc Bras Med Trop..

[73] Quadros RM, Marques S, Arruda AAR, Delfes PSWR, Medeiros IAA (2004). Parasitas intestinais em centros de educação infantil municipal de Lages, SC, Brasil. Rev Soc Bras Med Trop..

[74] Cimerman S, Cimerman B, Lewi DS (1999). Prevalence of intestinal parasitic infections in patients with acquired immunodeficiency syndrome in Brazil. Int J Infect Dis..

[75] Guimarães S, Sogayar MIL (1995). Ocurrence of *Giardia lamblia* in children of municipal day-care centers from Botucatu, São Paulo State, Brazil. Rev Inst Med Trop S Paulo..

[76] Kobayashi J, Hasegawa H, Forli AA, Nishimura NF, Yamanaka A, Shimabukuro T (1995). Prevalence of intestinal parasitic infection in five farms in Holambra, São Paulo, Brazil. Rev Inst Med Trop S Paulo..

[77] Guimarães S, Sogayar MI (1993). *Blastocystis hominis*: occurrence in children and staff members of municipal day-care centers from Botucatu, Sao Paulo State, Brazil. Mem Inst Oswaldo Cruz..

[78] Marques MVR, Ferreira Junior FC, Andery DA, Fernandes AA, Araújo AV, Resende JS (2013). Serologic, parasitic, and bacteriologic assessment of captive cracids (Aves: Galliformes: Cracidae) in Brazil. J Zoo Wildl Med..

[79] Marietto-Gonçalves GA, Fernandes TM, Silva RJ, Lopes RS, Andreatti-Filho RL (2008). Intestinal protozoan parasites with zoonotic potential in birds. Parasitol Res..

[80] Mundim MJS, Mundim AV, Santos ALQ, Cabral DD, Faria ESM, Moraes FM (2004). Helmintos e protozoários em fezes de javalis (*Sus scrofa scrofa*) criados em cativeiro. Arq Bras Med Vet Zootec..

[81] Brasil. Ministério das Cidades; Secretaria Nacional de Saneamento Ambiental - SNSA. Sistema Nacional de Informações sobre Saneamento: diagnóstico dos serviços de água e esgotos - 2014. Brasília: SNSA/MCIDADES; 2016.

[82] D’Avila R, Guerra EM, Rodrigues CI, Fernandes FA, Cadaval RA, Almeida FA (1999). Sobrevida de pacientes renais crônicos em diálise peritoneal e hemodiálise. J Bras Nefrol..

[83] Murad MH, Chu H, Lin L, Wang Z (2018). The effect of publication bias magnitude and direction on the certainty in evidence. BMJ Evid Based Med..

[84] Lau J, Ioannidis JP, Terrin N, Schmid CH, Olkin I (2006). The case of the misleading funnel plot. BMJ..

