# Supplementary material for: Prevalence of Blastocystis sp. infection in several hosts in Brazil: a systematic review and meta-analysis
Source: Parasit Vectors. 2020 Jan 14;13:30. doi: 10.1186/s13071-020-3900-2 (PMC6961275; doi:10.1186/s13071-020-3900-2)
Supplement: Supplementary file 1 — Additional file 1: Text S1. The search strategy. [file 13071_2020_3900_MOESM1_ESM.rtf]

Additional file 1: Text S1. The search strategy. Combination of mesh terms and keywords using Boolean operators.	1.  BVS(Blastocystis OR “Blastocystis sp”) AND (Hospedeiro OR Host OR Huésped) AND (Brasil OR Brazil)710 Results	2. Índice Bibliográfico Espanhol de Ciências da Saúde (IBECS)No results3. Literatura Latino-Americana e do Caribe em Ciências da Saúde (LILACS)227 results4. PUBMED (MedLine)(Blastocystis OR “Blastocystis sp”) AND (Host)139 results5. EMBASE(Blastocystis OR 'Blastocystis sp') AND Host185 results	6. CINAHL(Blastocystis OR 'Blastocystis sp') AND Host7 results7. WEB OF SCIENCE(Blastocystis OR “Blastocystis sp”) AND Host217 results	8. SCOPUS(Blastocystis OR “Blastocystis sp”) AND Host1.	1.	254 results9. BIBLIOTECA COCHRANEBlastocystis AND Host1 results
