# Supplementary material for: Prevalence of Blastocystis sp. infection in several hosts in Brazil: a systematic review and meta-analysis
Source: Parasit Vectors. 2020 Jan 14;13:30. doi: 10.1186/s13071-020-3900-2 (PMC6961275; doi:10.1186/s13071-020-3900-2)
Supplement: Supplementary file 3 — Additional file 3: Figure S1. The summary of methodological quality and bias risk and applicability for each study. [file 13071_2020_3900_MOESM3_ESM.pdf]

	Limitations in study design or execution (risk of bias)	Inconsistency of results	Indirectness of evidence	Imprecision	Publication bias
Aguiar et al. [67]	+	+	+	+	+
Alarcón et al. [68]	+	+	+	+	+
Amâncio et al. [57]	+	+	+	+	+
Amato-Neto et al. [72]	+	+	+	+	+
Batista et al. [59]	+	+	+	+	+
Borges et al. [63]	+	+	+	+	+
Branco et al. [58]	+	+	+	+	+
Cabrine-Santos et al. [47]	+	+	+	+	+
Carvalho-Costa et al. [69]	+	+	+	+	+
Cimerman et al. [74]	+	+	+	+	+
David et al. [9]	+	+	+	+	+
Eymael et al. [62]	+	+	+	+	+
Faria et al. [48]	+	+	+	+	+
Gil et al. [54]	+	+	+	+	+
Gil et al. [55]	+	+	+	+	+
Guimaraes et al. [75]	+	+	+	+	+
Guimaraes et al. [77]	+	+	+	+	+
Kobayashi et al. [76]	+	+	+	+	+
Kulik et al. [65]	+	+	+	+	+
Malheiros et al. [60]	+	+	+	+	+
Melo et al. [49]	+	+	+	+	+
Miné et al. [66]	+	+	+	+	+
Nascimento et al. [71]	+	+	+	+	+
Oliveira-Arbex et al. [45]	+	+	+	+	+
Quadros et al. [73]	+	+	+	+	+
Rebolla et al. [51]	+	+	+	+	+
Santos et al. [53]	−	+	+	−	−
Santos et al. [56]	+	+	+	+	+
Seguí et al. [46]	+	+	+	+	+
Seguí et al. [50]	+	+	+	+	+
Souza Júnior et al. [70]	+	+	+	+	+
Takizawa et al. [64]	+	+	+	+	+
Valença-Barbosa et al. [44]	+	+	+	+	+
Valença-Barbosa et al. [47]	+	+	+	+	+
Visser et al. [61]	+	+	+	+	+
