# Supplementary material for: Prevalence of Blastocystis sp. infection in several hosts in Brazil: a systematic review and meta-analysis
Source: Parasit Vectors. 2020 Jan 14;13:30. doi: 10.1186/s13071-020-3900-2 (PMC6961275; doi:10.1186/s13071-020-3900-2)
Supplement: Supplementary file 4 — Additional file 4: Figure S2. The summary of methodological quality and bias risk and applicability across the included studies. [file 13071_2020_3900_MOESM4_ESM.pdf]

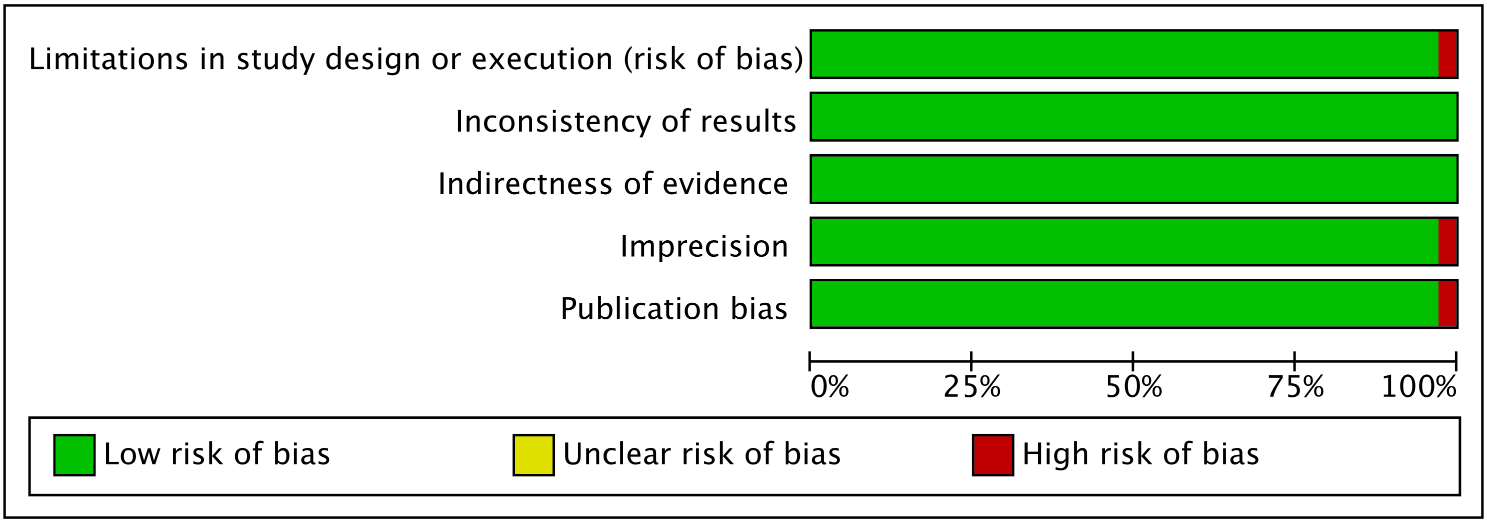

Limitations in study design or execution (risk of bias)
Inconsistency of results
Indirectness of evidence
Imprecision
Publication bias
0%
25%
50%
75%
100%
Low risk of bias
Unclear risk of bias
High risk of bias
